# Supplementary material for: Evolution of temperature preference behaviour among Drosophila larvae
Source: iScience. 2025 May 31;28(7):112809. doi: 10.1016/j.isci.2025.112809 (PMC12221525; doi:10.1016/j.isci.2025.112809)
Supplement: Document S1. Figures S1–S8 [file mmc1.pdf]

iScience, Volume *28*

Supplemental information

Evolution of temperature preference behaviour among *Drosophila* larvae

Tane Kafle, Manuel Grub, Panagiotis Sakagiannis, Martin Paul Nawrot, and J. Roman Arguello

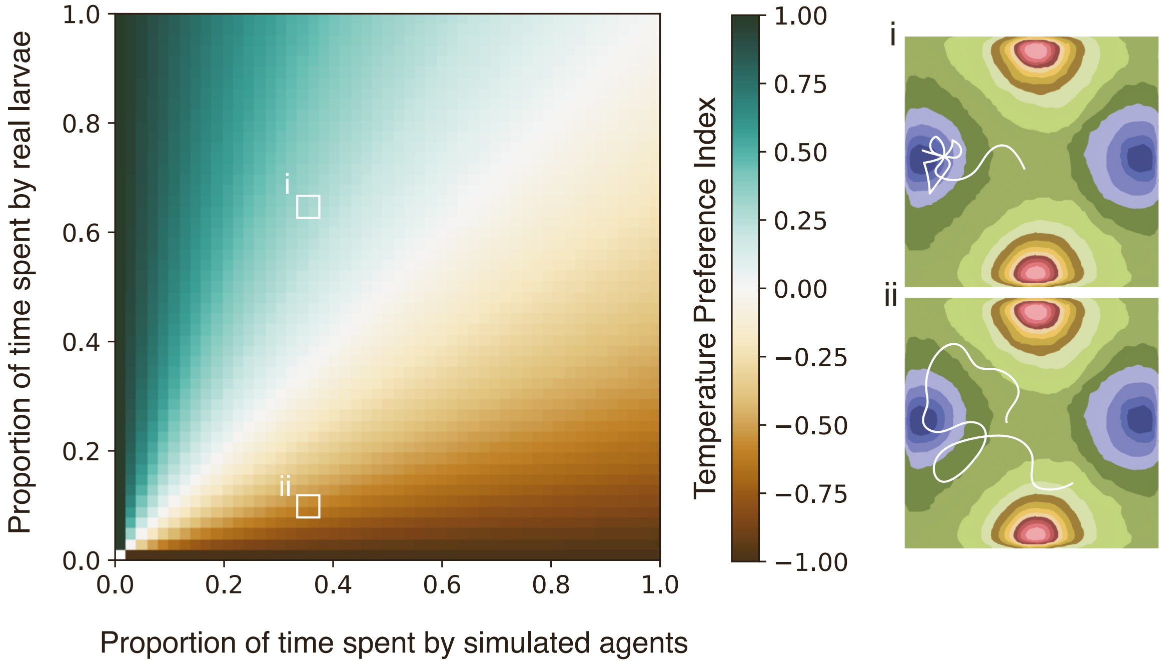

Figure S1 Influence of temperature bins on electivity values. The left panel is adapted from Lechowicz (1982). It illustrates the impact of temperature distribution within the arena, that affects both the proportion time spent by simulated agents and larvae on the arena, which ultimately impacts the final preference index values. As bins are unequal in size, randomly moving simulated agents will spend more time in larger bins, and this effects the electivity values possible for those temperature bins when accounting for the time larvae spend too. Also, as larvae are always not present in n-1 bins, values tend to be towards the lower numbers. Consequently, preference index values tend to be closer to zero and are often skewed towards negative values. Panels on the right represent example larval tracks, with the corresponding points on the left panel, to show how larval movement can affect preference index values, in this case, for the blue zone.

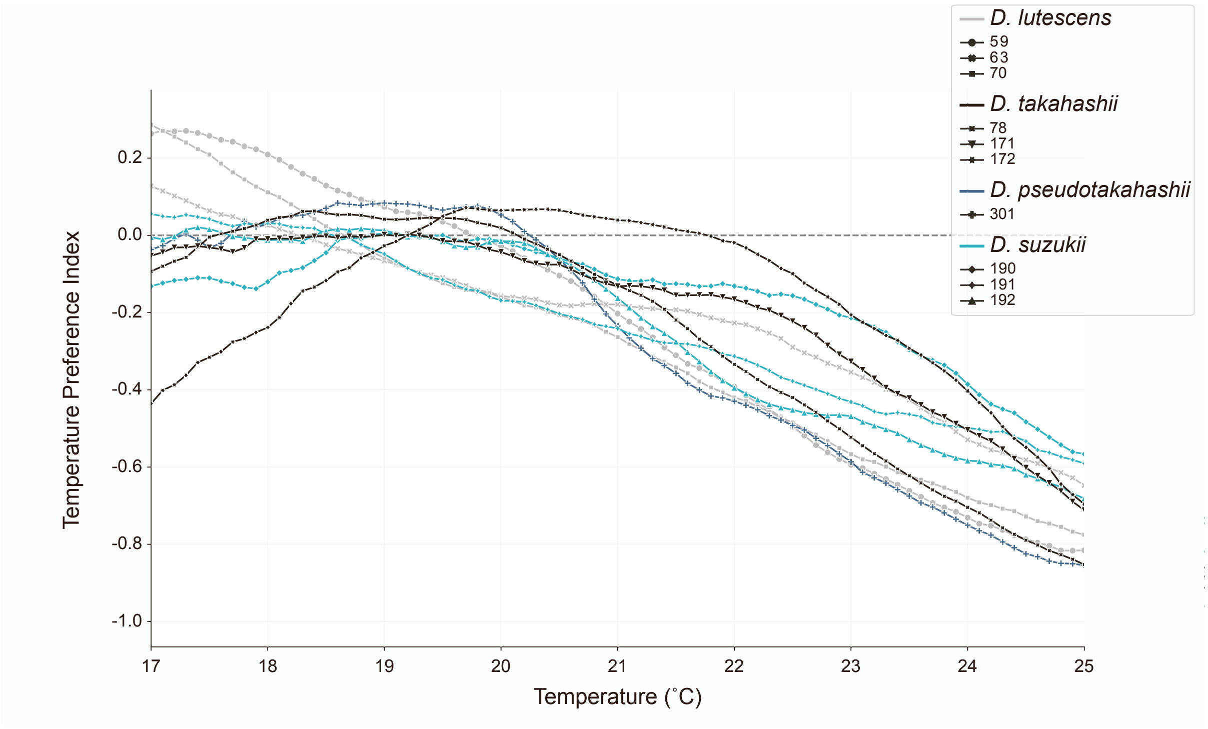

Figure S2 Electivity patterns across temperatures for different strains of the Oriental clade. The line plots illustrate the electivity patterns of strains used for each species across the range of temperatures. While there is variation among strains within a species, they often exhibit similar electivity patterns. *D. takahashii* 172 is a notable exception.

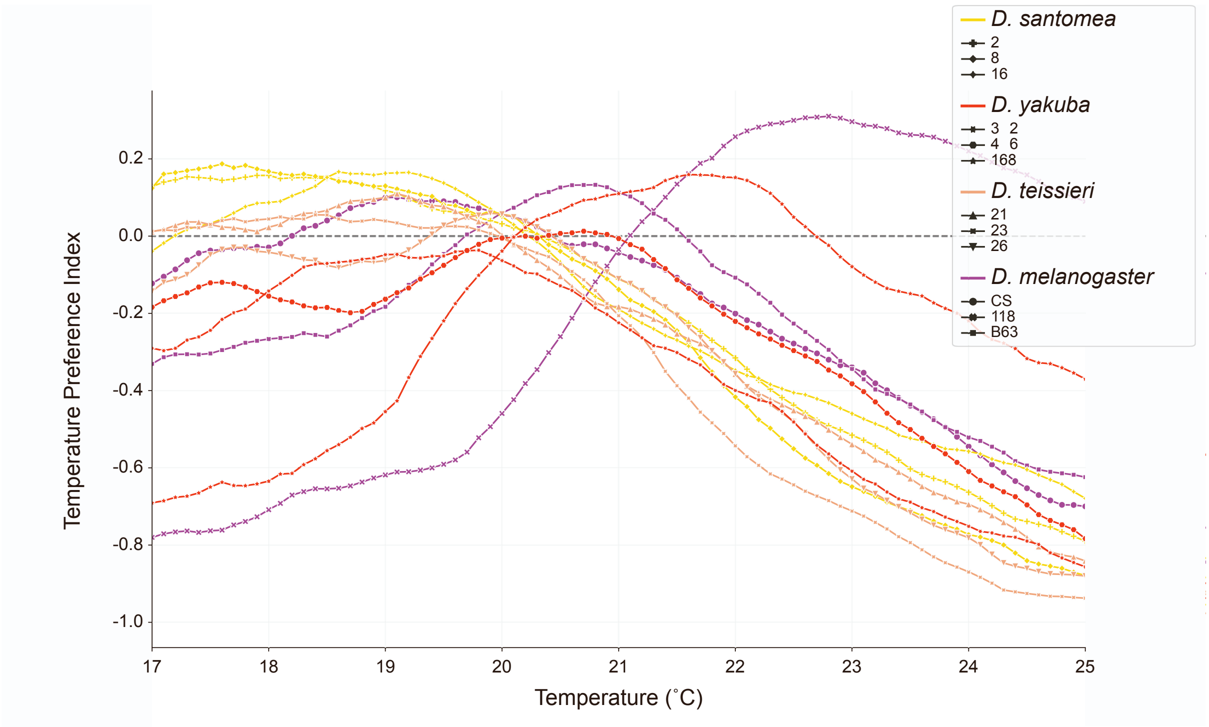

Figure S3 Electivity patterns across temperatures for different strains of the *Dmel*-subgroup. These line plots illustrate the electivity patterns of strains used for each species across the range of temperatures. While there is variation among strains within a species, they often exhibit similar electivity patterns. However, *D. melanogaster* w1118 and *D. yakuba* 168 deviate from their species' common profile.

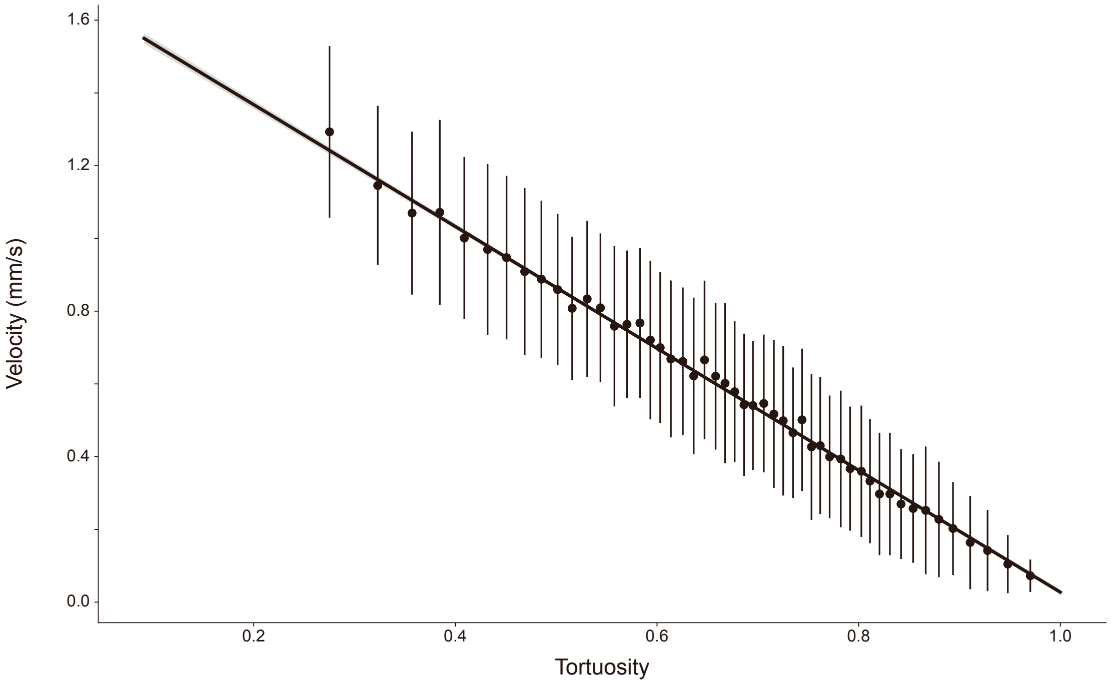

Figure S4 Correlation between velocity and tortuosity. This figure illustrates the negative relationship between velocity and tortuosity across all assays: the less tortuous (more straight) runs are associated with faster movement.

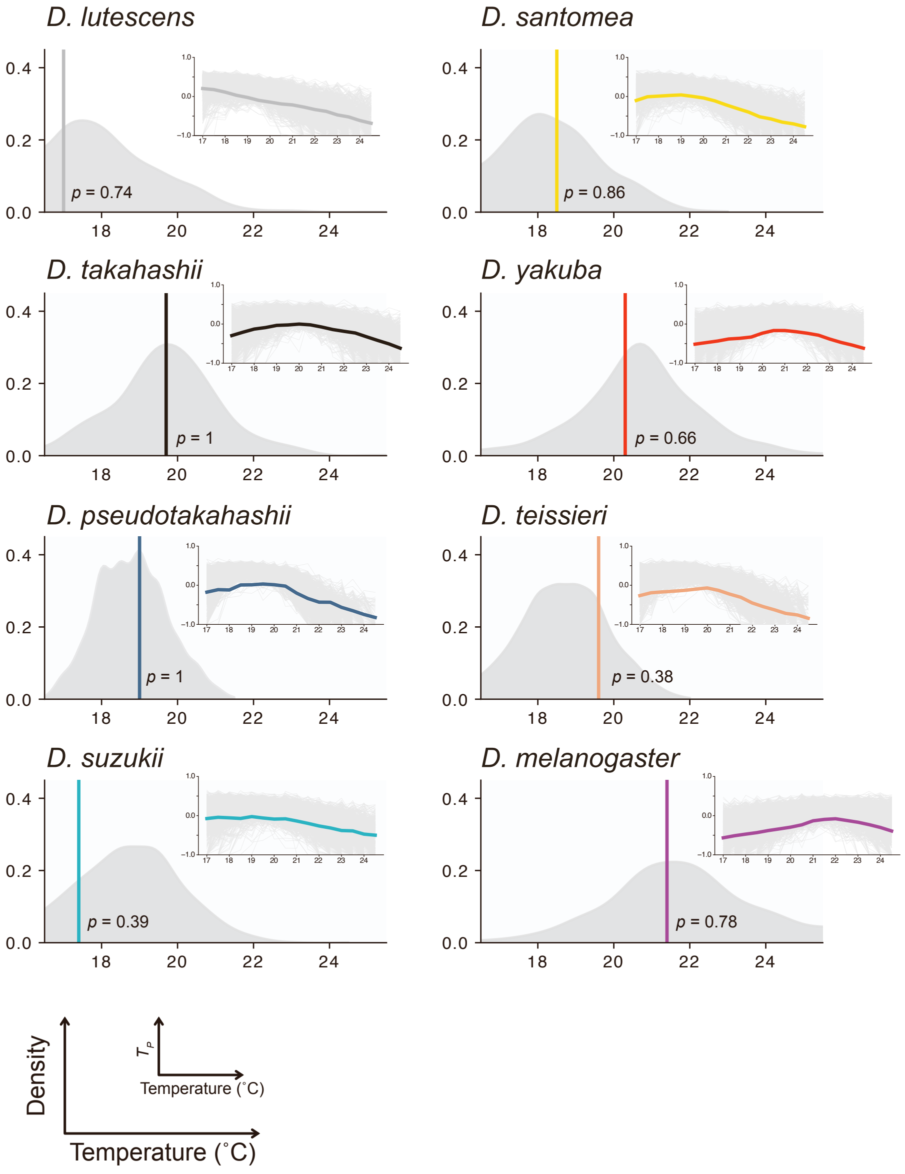

Figure S5 Result of predictive simulations based on the best fitting thermotaxis model for each species (see also Fig. 4). Each plot displays the distribution of Tp_{opt} resulting from 1000 simulations (grey) with the observed Tp_{opt} labelled with a coloured vertical line. *P*-values indicate the significance of the observed Tp_{opt} given the data generated from the model. The insets display the simulated 1000 *Tp* curves over temperature ranges in grey with the observed *Tp* curve shown as a coloured line.

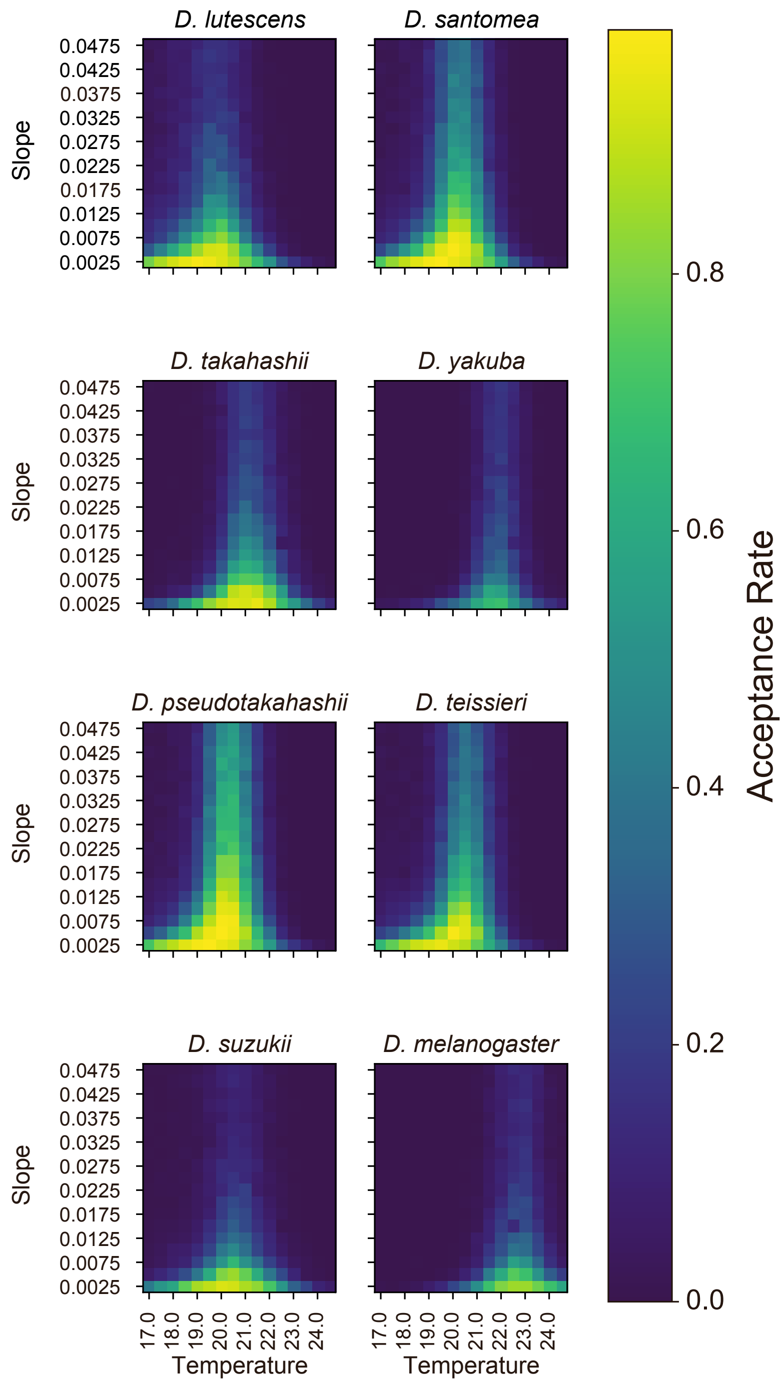

Figure S6 Preliminary grid search for optimal slope values. We used a course grid search to find a range of slope values for the final set of simulations. The results suggest that lower slope values were optimal for all species.

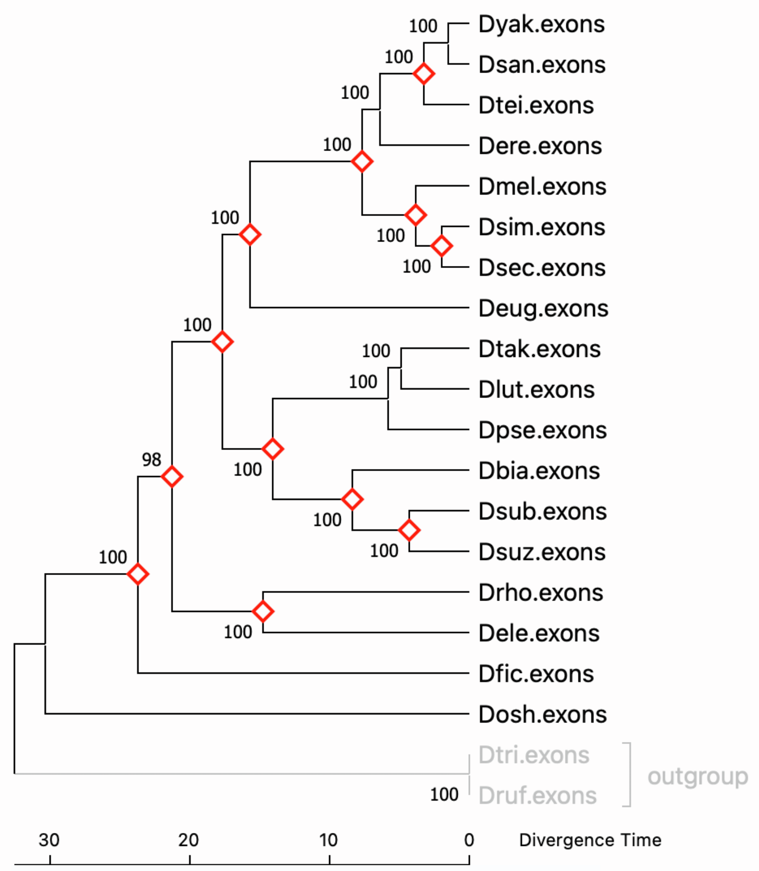

Figure S7 Dated phylogeny for species across the *D. melanogaster* subgroup and the Oriental clade. Red diamonds indicate dates that were input as calibration points from Table S5. The scale bar on the bottom displays the divergence time as measured in millions of years.

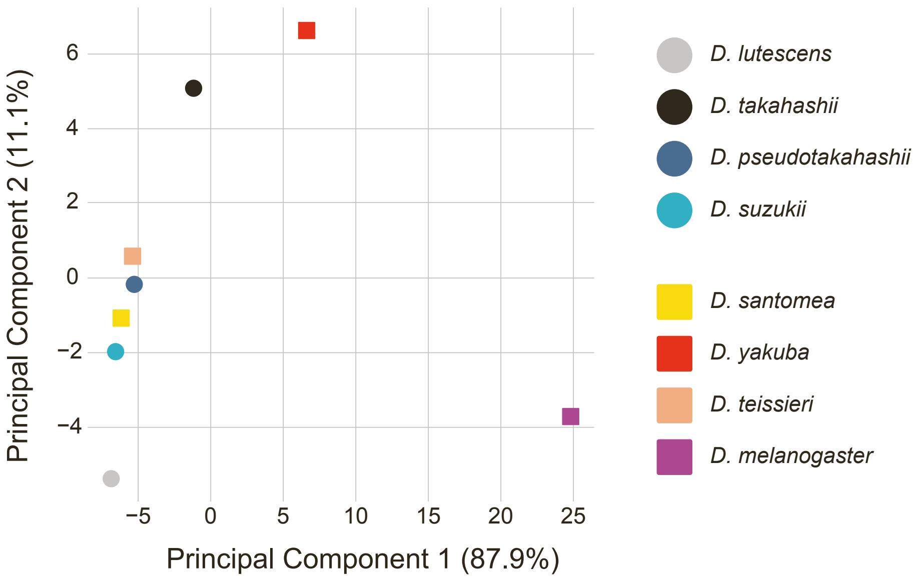

Figure S8 Results from a Principal Component Analysis that used the grid values for the best fitting simulations (white dots in Fig.4 A-H). The first axis separates *D. melanogaster* from all other species, with it having a homeostatic set point at higher temperatures than all other species. *Drosophila suzukii, D. pseudotakahashii*, *D. santomea* and *D. teissieri* sit very closely on both principal component axes, indicating that they have very similar fits to parameter sets.
